# Why There Is a Period to Life

**Published:** 1881-06

**Authors:** T. S. Sozinskey

**Affiliations:** Philadelphia.


### ARTICLE II.

### *Why There is a Period to Life.*

BY T. S. SOZINSKEY, M. D., PH. D. OF PHILADELPHIA.

That all living things, vegetable and animal, exist but for a limited period, is a truth familiar to every one. From the most lowly plant up to man himself all are doomed to pass away, to dissolve into the elements of inorganic creation. The law is universal; an exception to it is looked for in vain. Death awaits in common everything that is possessed of life.

Now, there are doubtless very few persons to whom has not occurred the question, why do we die? It is in every respect a legitimate one to ask, and we should at least try to furnish a satisfactory, a rational answer to it. That is what I propose to do briefly in this article.

Man has been curious to know why he is mortal, from the earliest times; and he was not slow to frame explanations of the fact. Like many other things which are very difficult to find out, or to comprehend, the very difficulty of the matter led early to its elucidation, in a way. Being beyond the easy ken of students of nature, it was taken hold of by students of theology, or rather elaborators of theology. An explanation of the origin or cause of death constitutes an important item of the scriptures of most religions; and it is essentially the same in all. Man is mortal in consequence of violating the command of a supernatural power; or, in other words, in consequence of sin. Thus St. Paul says, "By one man sin entered into the world, and death by sin; and so death passed upon all men, for that all have sinned." Of course, the eating of "the forbidden fruit" by Adam is here referred to.

Passing over similar ones, I may remark of the Mosaic explanation of man's mortality, that it has been and still is satisfactory to millions of people; but even if we take it as such, we are still in the dark as to the reason why other living things die. Human transgressions have had nothing to do with the origin of the mortality of other creatures; for death prevailed among them myriads of years before man appeared on the earth. The presence of their fossilized remains in the oldest strata of the earth's crust sufficiently attests the fact. Apart from other reasons this failure of the explanation to cover the whole ground impels one to doubt its validity, even in the case of man; and, in consequence, students of nature are forced to still occupy them selves with the problem.

It may be safely asserted that the real cause of death among living things, including man, is not undiscoverable. I believe that human knowledge has advanced far enough to enable us to correctly explain it. Every-day observation, as well as common sense, teaches us that there is a reason, a cause, for the death of everything; so it must be a subject which admits of scientific elucidation. The inductive method of study brought to bear on organic creation—which an enlightened philosophy teaches us is a systematic scheme —has cleared up so many difficulties which formerly seemed insuperable, that there are substantial grounds for confidence that this one will yield to it in the fullest degree.

Why is it, then, that we die? The reason is to be found in the fact that life is conditioned. Life is possible under certain conditions and impossible under others. Heat is necessary; so is air; and so is food. Without suitable heat and air and food, or any one of these, life cannot be. While the earth was in an incandescent state life did not exist thereon; because the heat was too great to admit of it. While the atmosphere was saturated with carbonic acid only plants existed; for when this gas is present in large amount it is destructive to animal life. And plants must have existed before animals, because the latter cannot draw sustenance directly from the inorganic world. Do we not see, then, that in understanding why we live, we at the same time get a clue to the reason why we die? While life is present death is not, and *vice versa*. To die is to cease to live.

Now, life being conditioned, of course, if the necessary conditions were wanting it could not exist.

I have just spoken of some of the necessary external conditions; but there are conditions of the organism as well as of the environment, which, if wanting, life cannot exist. Thus, the blood must circulate, the lungs must act, and the nerve-centres must have free play. In man, as in every one of the higher animals, anything which stops the functional action of the heart, or of the lungs, or of the brain, results in death. In organisms in which these organs are present in rudimentary, or specially modified form, the same result follows when they are interfered with, so that their functional activity is seriously perverted, or stopped. Between the internal and the external conditions there is a sort of antagonism. Herbert Spencer lays great emphasis on this antagonism; he goes so far as to regard life as "the continuous adjustment of internal relations to external relations." However, our argument leads us to the conclusion that it is inconceivable that an immortal being could exist in a world where the essential conditions of life are liable to be removed. If life were entirely independent of conditions it might continue for an indefinite length of time.

But how is it that age leads to death? Simply because the conditions necessary for the continuance of life are removed thereby. And we must search for the cause within the organism. The necessary external conditions still remaining, of course, the trouble must be seated in the internal conditions. Something must occur to the organism which results in the suspension of one or more functions necessary to life; because, as the medical philosopher Broussais puts it, "for a person to die, it is necessary that the principal instruments of life should be seriously injured." Some change is produced in the organism which interferes seriously with circulatory, or respiratory, or nervous action. And here I may say that if any one of the necessary functions is affected, all others suffer in consequence. The various parts and functions are intimately related to one another; each is, in a measure, dependent on all the rest for its welfare. From these premises it follows that death from age may be caused in different ways. In one person it may result through circulatory defects, in another, through respiratory defects, and in another, through nervous defects. To be sure, all these functions may be gradually encroached upon at the same time; and I imagine that in ideal death from old age such is the case.

Most physiologists hold that death from old age begins in the nervous system, in the brain. Bichat, the French genius who laid the foundation of modern physiology, in his immortal work on life and death, elaborated this belief. He came to the conclusion that the distinguishing features of animal life are merely additions to vegetable life. He says, "The functions of the animal are of two very different classes. By the one, which is composed of an habitual succession of assimilation and excretion, it lives within itself, transforms into its proper substance the particles of other bodies, and afterwards rejects them, when they are become heterogeneous to its nature. By the other it lives, it perceives, it reflects on its sensations, it moves according to their influence, and frequently is enabled to communicate its voice, its desires and its fears, its pleasures and its pains." The aggregate of the functions of the first order he named the organic life, and the aggregate of the second, the animal life. When death is typically natural, the animal life is cut off in advance of the organic life.

Death in man and in all animals begins in the central portion of the nervous system, in the brain. Dr. B. W. Richardson, whose views on this subject are essentially the same as Bichat's, in his work on diseases of modern life, says, "The end, when it comes at the duly and fully appointed time, is, I believe, always preceded by failure of the nervous stimulus. The muscles would continue to act if they were excited and directed into action; the secretions would proceed, and in truth often do proceed, when the faculties of the mind and of volition have ceased; but the nervous organism, through its varied parts, fails, and with it die all the parts that depend upon it for animation." I believe, as already stated, that from the dependence of the various functions on one another, the actual cause of the exhaustion of nervous energy or vital force may be located elsewhere than in the nervous system.

It used to be, and is still, the belief of some, that the essential cause of death from old age is the exhaustion of an inherited stock of vital force. One of Shakspeare's characters says of his life that it

> "Bleeds away, even as a form of wax

> Resolveth from its figure 'gainst the fire."

This opinion is untenable. Whatever store of vitality a person may have is constantly being added to as well as subtracted from; and it is in no wise necessarily lessening in its totality every succeeding day; on the contrary, it may be increasing. There is a constant renewal being effected; and herein lies the element of greatest difficulty in explaining why we die; for as Bacon says, "that which may be replaced by degrees without a total waste of the first stock is potentially eternal as the vestal fire."

While life lasts there is constant change going on in every organ of the body; old particles are constantly being replaced by new ones, which in their turn are replaced by others. This molecular change goes on at varying rates of speed in different organs and at different times. Some glands are replaced entirely in a few days, while bones change but slowly. Exercise hastens molecular activity or nutritive action in all the tissues; and various medicines known as stimulants and alteratives have a similar effect. It was formerly, and is still to some extent, the popular belief that the entire composition of the body is changed once every seven years. The nutritive activity is the fountain of life, the source of all vital phenomena; while it continues the organism is said to be alive, and when it stops, the organism is said to be dead.

From the beginning to the end of life it goes on incessantly. Does it not follow that death from a gradual running out of vitality results from the gradual stoppage of the nutritive activity? The power of repair fails, and when it ceases altogether dissolution sets in. As Dr. Rush, in his admirable essay on old age, says, "death from old age is the effect of a gradual palsy."

I believe, then, that the immediate cause of death from old age is exhaustion of vital force, or rather, of the power of reproduction of vital force. Says Dr. Carpenter, in his work on physiology, "Of this diminution of formative power we have evidence in the entire absence of any attempt at new development, in the less perfect and more tedious manner in which the losses of substance occasioned by disease or injury are recovered from, and in the gradual deterioration of the organism in general. The tissues which are rendered effete by their functional activity are not any longer replaced in their normal completeness; for either the quantity of new tissue is inadequate, so that the bulk of the organism is obviously reduced, or their quality is rendered imperfect by the production of structures in various phases of degeneration, in place of those which had been previously developed in the fullest completeness. The inferiority of nervo-muscular energy and of general vigor are thus evidently the result of the deficiency, and not, as in the period of growth, of the excess of formative power; and in proportion as the waste of the tissues consequent upon their functional activity is more rapid than their renovation, a progressive loss of substance must take place. The forms of degeneration most commonly met with in advanced age are the fatty and calcareous." Why this reparative power should fail, must, I believe, be sought for in structural change. Dr. Richardson believes he has found out what this change is. He says: "I have learned that the gradual transformation of the vital organs of the body from the advance of age is due to a change in the colloidal matter which forms the organic basis of all the living tissues. In its active state this substance is combined with water, by which its activity and flexibility are maintained in whatever organ it is present—brain, nerve, muscle, eyeball, cartilage, or membrane. In course of time this combination is lessened, whereupon the vital tissues become thickened, or to use the technical term, "pectous." By attraction of cohesion the organic particles are welded more closely together, until at length the nervous matter loses its mobility and the physical inertia is complete."

But I have not yet done more than hint at the reason, or reasons why we die. I have merely stated what occurs as death approaches, namely, that the tissues become more and more condensed and somewhat changed in substance, and that the vital power is gradually lessened. Just why these changes should occur has not been explicitly stated. Dr. Richardson, in discussing the subject, comes to the conclusion that "death is part of the scheme of life—is ordained upon a natural term of life." This statement is unsatisfactory to every questioning mind. We want to know something more; we want to know the reason why life passes away? why it is limited in date. I think this information can be furnished, and that the cycle of life need not be regarded as mysterious. The philosophy of why we die may be stated in a few words. It is a well established fact that in body and mind we reflect, in a great measure, the traits of our ancestors; or, in other words, for what we are in structure and function we are largely indebted to inheritance. At birth some of us are healthy, free from taints of disease, while others are just the reverse. Our parents have bequeathed to us bodies of various degrees of soundness. Those soundest to begin with are most likely to be the last to die. Now, passing through life we are constantly subjected to a great many influences which tend to destroy the vital spark. We have to contend with the elements, heat and cold, with mental dislikes, and so on. These influences which tend to destroy life are constantly contending with our vital powers, and when the former triumphs, destroys the latter, death is, of course, the result. This may come in a few days, or in a few months, or may be postponed for a hundred years; but it is the inevitable end of the struggle in every case. Let us foster and fortify our vitality as we may, it will give out. Extreme age is simply the longest period that life can hold out against the physical forces which are constantly operating to destroy it. Changes of structure are due to vicissitudes of vital action, and these lead gradually to impairment in the power of renewal of the latter. Such is, in brief, the explanation of why we die.—*Med. and Surg. Reporter.*

---

